# Clinical and radiographic evaluation of the Periodontium with biologic width invasion

**DOI:** 10.1186/s12903-020-01101-x

**Published:** 2020-04-16

**Authors:** Bruna Almeida Silva Carvalho, César Augusto Barroso Duarte, Jaciara Fagundes Silva, Walter Winícius da Silva Batista, Dhelfeson Willya Douglas-de-Oliveira, Evandro Silveira de Oliveira, Luana de Goés Soares, Endi Lanza Galvão, Gabriela Rocha-Gomes, José Cristiano Ramos Glória, Patrícia Furtado Gonçalves, Olga Dumont Flecha

**Affiliations:** Department of Dentistry, Federal University of Jequitinhonha and Mucuri Valleys, Rua da Glória, n° 187. Centro, Diamantina, MG 39100-000 Brazil

**Keywords:** Biologic width, Periodontal health, Gingival inflammation, Bone loss, Interproximal radiography

## Abstract

**Background:**

The biologic width is defined as the coronal dimension to the alveolar bone that is occupied by healthy gingival tissue. The objective of the present study was to correlate radiographic findings of biologic width invasion with the periodontium status.

**Methods:**

It were included 14 patients with restored teeth with biological width invasion, on the proximal sites, observed clinically and radiographically. 122 proximal sites were evaluated, 61 in the test group (biological width invasion) and 61 in the control group (adequate biological width). Smokers and patients presenting periodontal disease or restorations with contact in eccentric movements, horizontal over-contour or secondary caries were excluded from the sample. The invasion of the biologic width was diagnosed when the distance from the gingival margin of restoration to the bony crest was less than 3 mm. Intrabony defect and bone crest level, as well as, their vertical and horizontal components were radiographically evaluated when present. Plaque index, bleeding on probing, probing depth, gingival recession height, keratinized gingival height and thickness, and clinical attachment level were clinically evaluated. Data were subjected to Spearman’s Correlation and Wilcoxon’s test.

**Result:**

The most prevalent tooth with biological width invasion was the first molar. There was a statistically significant correlation between the bone crest (*p* < 0.001), vertical (*p* < 0.001) and horizontal (*p* = 0.001) components. In the test group, there was a statistically significant correlation between bleeding on probing (*p* < 0.001; *r* = 0.618) and width of gingival recession (*p* = 0.030; *r* = − 0.602) with the intraosseous component; and between keratinized gingival height and bone level (*p* = 0.037; *r* = − 0.267). In the control group, there was a correlation between plaque index (*p* = 0.027; *r* = − 0.283) with bone level and correlation between keratinized gingival thickness and bone level (*p* = 0.034; *r* = − 0.273) and intrabony component (*p* = 0.042; *r* = 0.226).

**Conclusion:**

A statistically significant relationship was found between bleeding on probing and gingival recession in patients who presented intrabony defects due to the invasion of biological width, which may be also related to the thickness of the keratinized gingiva.

## Background

The supracrestal gingival tissue, also known as the biologic width is defined as the dimension that the healthy gingival tissue occupies coronally to the alveolar bone, involving the sum of the junctional epithelium and the connective insertion dimensions [1–3]. The average vertical dimensions of the biological width are known thanks to the study of Gargiulo et al. (1961) [4], however, nowadays, it is known that these measurements are not constant, being dependent on the location/inclination of the tooth in the socket [5, 6], varying between teeth [7], their sites [8] and gingival biotypes [9]. In general, its length is 3 mm from the bone crest to the cement-enamel junction in healthy teeth or until the end of the preparation or the margin of restoration in restored teeth [10].

The existence of the biologic width is fundamental for adhesion of the junctional epithelium and insertion of the connective fibers to the dental structure, besides functioning as a barrier against microbial entry in the periodontium. Therefore, the biologic width should be respected during restorable procedures in order to preserve periodontal health [11, 12].

Biologic width invasion may cause injuries to periodontal tissues, as a means of maintaining its physiological dimensions, resulting in chronic inflammation of the soft tissues around the restoration, bleeding on probing, gingival hyperplasia, gingival recession, periodontal pocket, with loss of clinical insertion and progressive alveolar bone loss, in addition to difficulties in adapting restorations [13, 14].

When a patient is evaluated with a periodontal probe and feels discomfort in the gums, close to a restoration, it may suggest that the margin violated the biologic width. The determination of the dimension of supracrestral gingival tissues through transulcular periodontal probing, as described by Jardini and Pustiglione [7], has been shown to be an important auxiliary method in the diagnosis of biological width violation [15]. The interproximal radiography, in turn, can identify these violations and is considered the ideal technique for a more accurate assessment of the proximal sites, in addition to being a non-invasive method [14, 16].

There is a gap in the literature about radiographic and clinical comparison in cases of biologic width invasion. An adequate understanding of the relationship between clinical and radiographic findings is necessary for a better diagnosed and treatment. The aim of this study was to correlate radiographic findings with the clinical conditions of periodontium in sites presenting biologic width invasion, in cases of both direct and indirect restorations.

## Methods

This cross-sectional study was approved by the Research Ethics Committee of the Federal University of Jequitinhonha and Mucuri Valleys (UFVJM), by protocol 026/12 and conducted according to the Declaration of Helsinki, 1975, revised in 2013. Written informed consent was obtained from all participants prior the study begins.

### Patient selection

Patients who attended at the clinics of the Department of Dentistry UFVJM were invited to participate in this research until complete the sample. They were clarified about the objective, risks and benefits of participating in the research.

The sample size was determined with the following parameters: significance level 95%, power of 80%, minimum difference to be detected between the groups of 0.1 mm, and the standard deviation of probing depth (0.18 mm) was obtained in a previous study [17]. It were added 20% to prevent losses. The calculations have determined that 61 teeth sites with invasion of biologic width would be sufficient to carry out the study.

### Eligibility criteria

The study included patients over 18 years of age, with good general health, with no distinction of gender, ethnicity or socioeconomic status. It were included restored teeth with biologic width invasion, on the mesial or distal sites, which was radiographically observed. It was considered as biologic width invasion when the distance between the restoration gingival margin and the alveolar bone crest was less than 3 mm [18].

Patients presenting periodontal disease, smokers or who had restorations with contact in eccentric movements, horizontal overcontour or secondary caries were excluded from the sample.

### Radiographic evaluation

**Fig. 1:**
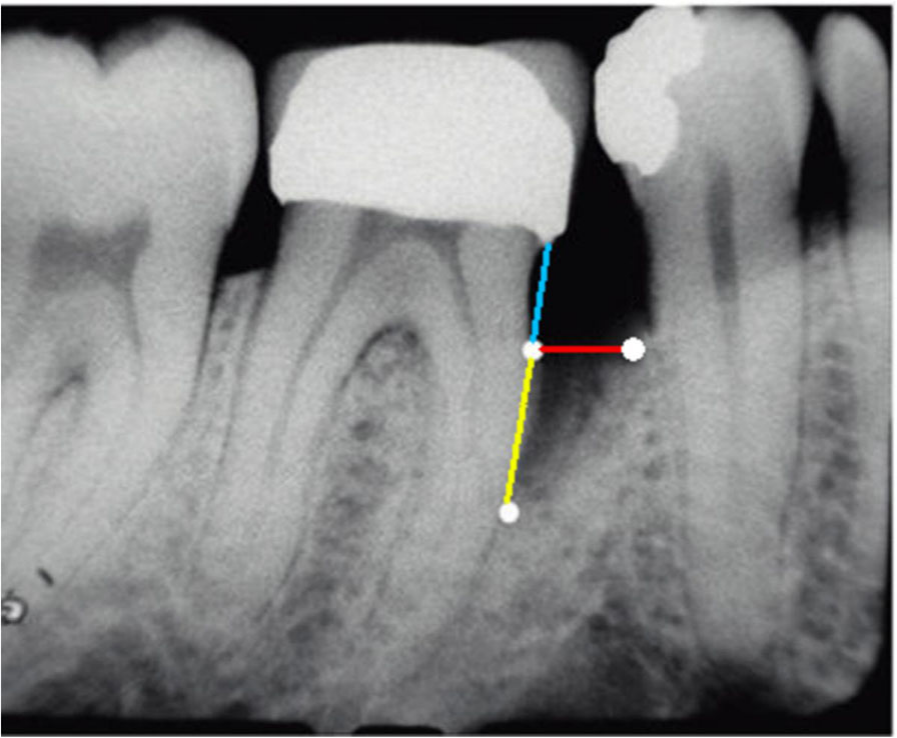
(Adapted from Parashis et al., 2012). Radiographic parameters evaluated in intrabony defect. Blue: BCL; yellow: VC; red: HC; blue+yellow: BDL

The radiographic parameters evaluated, in the test (biological width invasion) and control groups (adequate biological width), when there was intrabony defect were: (1) bone defect level (BDL), which is the distance between the restoration margin and the most apical portion of the defect where the space of the periodontal ligament presented normal width; (2) Bone crest level (BCL), measured between the margin of restoration and the projection of the most prominent portion of the alveolar bone crest on the root surface; (3) Vertical component (VC), defined by subtraction BDL - BCL; and, (4) horizontal component (HC), distance from bone crest to root surface (Fig. 1). The radiographs analyzed were those that were already present in the patient records who were attended at the UFVJM dental clinic.

These measurements were taken with a dry-tip compass and transformed in millimeters with the aid of a millimeter ruler, for greater accuracy and reliability, being obtained by a second duly calibrated researcher.

The sites with biological width invasion were considered test group. As control group, it were used sites of the analogous or antagonistic tooth to the one that presented biologic width invasion.

### Clinical evaluation

The clinical parameters evaluated, in the test and control groups, were: plaque index (PI) and bleeding on probing (BOP), both taken in percentage in relation to total number of sites, considering as positive the sites with visible plaque and with bleeding until 15 s after probing. The probing depth (PD) was determined by the distance from the gingival margin to the base of the gingival sulcus, performed with a computerized periodontal probe (Florida Probe). The gingival recession height (GRH) consists of the distance from the cement-enamel junction to the most apical extension of the gingival margin; gingival recession width (GRW), given by the distance from the right gingival margin to the left gingival margin at cement-enamel junction. Keratinized gingiva height (KGH), measured as the distance from the gingival margin to the mucogingival line. Clinical attachment loss (CAL) given by the sum of PD and GRH. The keratinized gingiva thickness (KGT) was measured by perforation of the gingival tissue with a thin digital endodontic spacer and a rubber stop in the half of the KGH under topical anesthesia. The GRH, GRW and KGH were taken with manual periodontal probe and measured in millimeters. The clinical evaluation was performed by four examiners (BASC, CABD, JFS and WWSB) previously calibrated, the inter-examiner ICC ranged from 0.799 to 0.890 and the intra-examiner ICC ranged from 0.842 to 0.903.

### Statistical analysis

Statistical analyses were performed using the SPSS® (Statistical Package for the Social Sciences Inc.), version 22. Descriptive analysis of the data provided frequencies, means and standard deviations. Data normality was assessed by the Shapiro-Wilk test. The Spearman correlation and Wilcoxon test were used to verify, respectively, the correlation and the association between the clinical and radiographic parameters. The confidence interval of 95% was used, and the significance level adopted was 5%.

## Results

The sample consisted of 13 women (93%) and 1 man (7%). The tooth that presented most biologic width invasion was the first molar, in both groups. It were evaluated 122 sites, being 30 mesial (49%) and 31 distal (51%) sites in test group, and 29 mesial (47.5%) and 32 distal (52.5%) sites in the control group (Table 1). Gingival recession was observed in 18 teeth in test group and in 5 teeth in control group.

**Table 1:** Prevalence of biologic width invasion, according to teeth, to dental site and to the group

Parameter	Test group		Control group	
	n	%	n	%
**Tooth**				
First Molar	19	31.1	18	29.5
Second Molar	8	13.1	11	18.0
First Pre-molar	11	18.0	10	16.4
Second Pre-molar	18	29.5	16	26.2
Canine	3	4.9	4	6.6
Incisor	2	3.3	2	3.3
**Site**				
Mesial	30	49.2	29	47.5
Distal	31	50.8	32	52.5

Table 2 shows the descriptive statistics for test and control groups. A statistically significant difference was observed between bone crest level (*p* < 0.001), bone defect level (*p* = 0,005), vertical (*p* < 0.001) and horizontal components (*p* = 0.001).

**Table 2:** Average of the parameters related to the sites probed

	Test group		Control group		
Parameters related to the sites probed	Mean	SD	Mean	SD	*p*-value
Probing depth	2.5	1.4	2.1	1.1	0.080
Clinical attachment level	2.7	1.4	2.3	1.3	0.095
Height of gingival recession	2.3	1.0	2.3	0.6	0.655
Width of gingival recession	4.0	1.6	2.7	1.3	0.180
Keratinized gingiva height	3.2	1.7	2.7	0.6	0.089
Keratinized gingiva thickness	1.4	0.4	1.4	0.6	0.691
Level of bone defect	0.9	1.3	0.2	0.9	**0.005**
Bone crest level	1.4	0.6	2.4	1.2	**< 0.001**
Intrabony component	0.6	0.7	0.1	0.2	**< 0.001**
Horizontal component	0.3	0.5	0.1	0.2	**0.001**

Values in bold showing statistically significant difference (*p* < 0.05).

The correlation between the clinical and radiographic parameters of the test group is observed in Table 3. In test group, it was observed a statistically significant correlation between BOP with the intraosseous component (*p* < 0.001) (*r* = 0.618), and GRW with the intraosseous component (*p* = 0.030) (*r* = − 0.602); and, between the KGH and the bone level (*p* = 0.037) (*r* = − 0.267). In the control group, the correlation occurred between PI and BCL (*p* = 0.027) (*r* = − 0.283); and between KGT and BCL (*p* = 0.034) (*r* = − 0.273) and also of the intraosseous component (*p* = 0.042) (*r* = 0.226), (Table 4).

**Table 3:** Correlation of clinical and radiographic findings for test group

Clinical parameters	Radiographic parameters							
	Level of bone defect		Bone crest level		Intrabony component		Horizontal component	
	r_s_	*p*	r_s_	p	r_s_	*p*	r_s_	*p*
Plaque index	0.025	0.850	0.184	0.156	0.032	0.845	0.062	0.635
Bleeding on probing	−0.013	0.919	−0.012	0.929	0.618	**< 0.001**	0.001	0.991
Probing depth	−0.010	0.940	−0.035	0.788	−0.037	0.821	0.033	0.802
Clinical attachment level	0.131	0.315	−0.084	0.519	0.294	0.069	0.128	0.325
Height of gingival recession	0.271	0.277	−0.104	0.682	0.355	0.234	0.420	0.083
Width of gingival revession	−0.127	0.616	0.292	0.240	−0.602	**0.030**	−0.266	0.287
Keratinized gingiva height	−0.267	**0.037**	−0.069	0.595	0.034	0.840	−0.266	0.080
Keratinized gingiva thickness	−0.072	0.580	0.101	0.436	−0.172	0.294	−0.072	0.580

Values in bold showing statistically significant difference (*p* < 0.05).

**Table 4:** Correlation of clinical and radiographic findings for control group

Clinical parameters	Radiographic parameters							
	Level of bone defect		Bone crest level		Intrabony component		Horizontal component	
	r_s_	*p*	r_s_	p	r_s_	*p*	r_s_	*p*
Plaque index	0.166	0.201	−0.283	**0.027**	0.156	0.231	0.231	0.073
Bleeding on probing	−0.122	0.348	−0.063	0.629	−0.131	0.314	−0.014	0.915
Probing depth	−0.107	0.411	0.222	0.086	−0.123	0.343	0.100	0.444
Clinical attachment level	−0.126	0.333	0.233	0.071	−0.141	0.277	0,075	0.564
Keratinized gingiva height	−0.065	0.620	0.088	0.498	−0.080	0.541	0.101	0.438
Keratinized gingiva thickness	0.247	0.055	−0.273	**0.034**	0.262	**0.042**	0.052	0.688

The height and width of gingival recession did not run in this correlation due the low sample. Values in bold showing statistically significant difference (*p* < 0.05).

### Discussion

Periodontal health is a basic requirement for both the longevity of restoration and the aesthetics, as well as, function and maintenance of dentition. However, dental restorations presenting width invasion are a frequently problem in clinical practice and are capable of inducing gingival inflammation, loss of connective tissue and unpredictable bone loss [19, 20]. Also, the invasion of the biologic width may cause periodontal pocket which does not imply the diagnosis of periodontal disease.

It was observed that the first molar, in the test and control groups, showed greater invasion of biological space. According to Vacek et al. [5], there are variations in the dimensions of the supracrestal gingival tissue between teeth, as well as in their sites, with the average molar measurements being greater than in the other groups of teeth.

The gingival recession and inflammation were clinically observed in this study, in addition to the correlation between the presence of width invasion and the decrease in the level of the bone crest observed radiographically, these findings similar to those reported by Douglas-de-Oliveira et al. [17].

The relationship between width invasion and bleeding on probing found in the literature and in the present study can be explained by the fact that the placement of restorative margins within the width space often leads to gingival inflammation, loss of clinical attachment and bone loss. This is probably due to the destructive inflammatory response of the microbial located deeper into the gingival sulcus. These alterations were justified by studies that evaluated the histological and clinical response of periodontal tissues to the position of the restoration margins within the biologic width [21, 22].

In the present study, the negative correlation between keratinized tissue height and bone level was observed in the test group, which means that the higher the keratinized tissue, the lower the level of bone defect. In fact, according to Stetler and Bissada [23], teeth with subgingival restorations and narrow keratinized gingiva have worse gingival inflammation compared to a wide range of keratinized tissue.

A negative correlation was found between plaque index and bone crest level in teeth with biologic width invasion in the control group. This can happen due to the biofilm retention in the rough surface areas of restoration that was brought into the gingival sulcus, where the patient is unable to properly cleaning his/her tooth, aggravating biofilm accumulation [24]. Consequently, this condition could lead to progressive gingival inflammation followed by periodontal destruction with greater pocket depth, attachment loss and gingival recession, increasing vertical bone resorption and increasing the horizontal component [17].

A negative correlation was also found between the thickness of the keratinized gingiva and the level of the bone crest in the control group. This result is in agreement with studies that evaluated gingival phenotype [9] in which a greater distance from the supracrestal gingival tissue was found in thin phenotype compared to the thick phenotype.

Thus the use of radiographic data is important to diagnose the biologic width invasion. Interproximal radiographs are the most used one for this purpose, since it presents less distortion than the other techniques [25]. In addition to this technique, a recent study [26] has shown an innovative parallel-profile radiography technique for gauging the dimensions of biological space on the labial sites of anterior teeth.

In a patient with preserved biologic width, the measure up to the bone crest has an average of 3 mm [12]. Furthermore, in the comparison between clinical and radiographic parameters in patients with invasion of the biologic width, association was observed between bleeding on probing, gingival recession and bone defects. This association was not found in the teeth of the control group, confirming the deleterious aspect of overlap restorations. These findings corroborate the concept that apically placed restorations within the supracrestal connective tissues may be harmful to periodontal health [27, 28].

## Conclusion

It was concluded that the restorations with biologic width invasion were harmful to periodontal health, showed a statistically significant relationship between the bleeding on probing and gingival recession in those patients who had intrabony defect.

According to the results of the present study, it is suggested that the clinician should consider the gingival phenotype, as well as, bone morphotype, when performing restorative procedures in areas near the gingival sulcus.

To try to minimize these damages, dentists should respect the measurements of the biologic width in direct and indirect subgingival restorations, since the health of the periodontium depends on the non-violation of this anatomic site.

## Data Availability

The datasets used and/or analysed during the current study are available from the corresponding author on reasonable request.

## Abbreviations

BCL: Bone Crest Level
BDL: Bone Defect Level
BOP: Bleeding On Probing
CAL: Clinical Attachment Loss
GRH: Gingival Recession Height
GRW: Gingival Recession Width
HC: Horizontal Component
KGH: Keratinized Gingiva Height
KGT: Keratinized Gingiva Thickness
PD: Probing Depth
PI: Plaque Index
VC: Vertical Component
